# Effects of Sleep on Word Pair Memory in Children – Separating Item and Source Memory Aspects

**DOI:** 10.3389/fpsyg.2017.01533

**Published:** 2017-09-07

**Authors:** Jing-Yi Wang, Frederik D. Weber, Katharina Zinke, Hannes Noack, Jan Born

**Affiliations:** ^1^Institute of Medical Psychology and Behavioral Neurobiology, University of Tübingen Tübingen, Germany; ^2^State Key Laboratory of Cognitive Neuroscience and Learning & IDG/McGovern Institute for Brain Research, Beijing Normal University Beijing, China; ^3^Graduate School of Neural and Behavioral Sciences, University of Tübingen Tübingen, Germany; ^4^Department of Psychiatry and Psychotherapy, University of Tübingen Tübingen, Germany; ^5^Werner Reichardt Center for Integrative Neuroscience, University of Tübingen Tübingen, Germany

**Keywords:** memory consolidation, electroencephalography, declarative memory, episodic memory, child development, sleep

## Abstract

Word paired-associate learning is a well-established task to demonstrate sleep-dependent memory consolidation in adults as well as children. Sleep has also been proposed to benefit episodic features of memory, i.e., a memory for an event (item) bound into the spatiotemporal context it has been experienced in (source). We aimed to explore if sleep enhances word pair memory in children by strengthening the episodic features of the memory, in particular. Sixty-one children (8–12 years) studied two lists of word pairs with 1 h in between. Retrieval testing comprised cued recall of the target word of each word pair (item memory) and recalling in which list the word pair had appeared in (source memory). Retrieval was tested either after 1 h (short retention interval) or after 11 h, with this long retention interval covering either nocturnal sleep or daytime wakefulness. Compared with the wake interval, sleep enhanced separate recall of both word pairs and the lists *per se*, while recall of the combination of the word pair and the list it had appeared in remained unaffected by sleep. An additional comparison with adult controls (*n* = 37) suggested that item-source bound memory (combined recall of word pair and list) is generally diminished in children. Our results argue against the view that the sleep-induced enhancement in paired-associate learning in children is a consequence of sleep specifically enhancing the episodic features of the memory representation. On the contrary, sleep in children might strengthen item and source representations in isolation, while leaving the episodic memory representations (item-source binding) unaffected.

## Introduction

Sleep facilitates memory consolidation with ample evidence, especially for declarative memories (Rasch and Born, 2013). Many of these studies have employed the declarative word paired-associate learning task. In this task, subjects study a list of associated word pairs and are asked to recall the word pairs after a retention interval by presenting the first word of each pair. Sleep compared to wakefulness after learning robustly enhances memory for the studied pairs, in adults (Plihal and Born, 1997; Payne et al., 2012) and children (Backhaus et al., 2008; Wilhelm et al., 2008; Potkin and Bunney, 2012).

The beneficial effect of sleep on declarative memory consolidation has been assumed to rely on a process of system consolidation involving neural reactivations that primarily affect the episodic features of a memory representation residing in hippocampal networks (Diekelmann and Born, 2010; Inostroza and Born, 2013). Specifically, the hippocampus is thought to encode an episode by binding an event (item) into its spatiotemporal context (source). Thus, memory for episodic features, such as information about when and where an event occurred crucially relies on the hippocampus (Lehn et al., 2009; Devito and Eichenbaum, 2011), and memory for such contextual information seems to be supported by sleep (Drosopoulos et al., 2007; van der Helm et al., 2011). Moreover, sleep also appears to support the binding of item memory into source memory which is characteristic for episodic memory (Inostroza et al., 2013; Oyanedel et al., 2014; Weber et al., 2014), although other studies show the opposite, i.e., a ‘de-contextualizing’ effect of post-encoding sleep enhancing the unbinding of episodic memory such that the memory for items becomes less dependent on the spatiotemporal source in which it was learned (Cairney et al., 2011; Deliens and Peigneux, 2014; but see Sweegers and Talamini, 2014; Jurewicz et al., 2016, for conflicting evidence).

Children show robust abilities to form memories for events (items) early during development (Mullally and Maguire, 2014). However, memory for source information, like the spatial and temporal context an event has occurred in, shows a protracted trajectory of development throughout the first decade of life and even beyond (Bauer and Lukowski, 2010; Picard et al., 2012) with a distinct developmental trajectory for the binding of item and source (Riggins, 2014). This slow development appears to be partly due to the protracted maturation of the brain structures involved in episodic memory formation (Gogtay et al., 2006; Ghetti and Bunge, 2012). However, children’s sleep is also longer and deeper, with higher proportions of slow-wave sleep (SWS) containing more intense slow-wave activity, reaching a maximum in pre-adolescence (Ohayon et al., 2004; Huber and Born, 2014; Wilhelm et al., 2014). Because processes during SWS such as slow-wave activity and associated spindle activity, are implicated in the consolidation of declarative memory (e.g., Marshall et al., 2006; Ngo et al., 2013), children might be expected to display enhanced sleep-dependent memory consolidation, despite a less developed episodic memory system and less prior knowledge (James et al., 2017).

Against this backdrop, our study aimed to dissociate to what extent the enhancing effect of sleep on word pair memory in 8- to 12-year-old children might originate from sleep strengthening the episodic features of the memory, in particular. To this end, we modified a classical paired-associates learning task known to benefit from sleep in children, such that participants now learned two lists (source) of semantically related word pairs (items), which were separated by a 1-h interval. At cued recall the participants received a single word as a cue and were asked to recall the other word in the pair as well as the list (List 1 or List 2) from which that pair originated. This procedure allowed us to contrast word pair memory in isolation (item memory), list memory associated with the cue word (source memory), as well as the combination of these two aspects (episodic memory). If the effect of sleep on word pair memory is mediated by strengthening the episodic memory aspects, we expected to find stronger episodic memory (i.e., correct combined word pair and list recall) specifically after sleep compared to wakefulness. Additionally, we expected that time spent in SWS would predict better retention of the combined word pair and list recall, whereas spindle activity may be associated with item memory, independently of whether it is bound or unbound to a source.

To compare the dynamics of memory retention between children and adults, we additionally included a sample of adults. Based on previous literature on age differences in episodic memory, we expected adults to generally outperform children in episodic memory (combined word pair and list recall). If sleep (and particularly SWS) strengthens episodic memory in particular, we expected to see diminished age differences in episodic memory after sleep because children show more SWS compared to their adult counterparts.

## Materials and Methods

### Participants

Sixty-one healthy children (8–12 years) without any known neurological or psychiatric disorders were recruited from local schools. Two children had to be excluded because of missing data, and one for taking a nap during the wake condition. Participants were assigned to three experimental groups with age and gender balanced: one group that slept for a whole night during a long retention interval between encoding and retrieval phase (Sleep group: 9.9 ± 0.24 years, *n* = 21, 10 males), another group that had a day awake during a long retention interval (Wake group: 10.1 ± 0.28 years, *n* = 17, 10 males), and one with a short retention interval to estimate retrieval shortly after encoding before going to bed (Pre-Sleep: 9.65 ± 0.27 years, *n* = 20, 9 males). To compare the dynamics of memory retention between children and adults, an additional control sample of 37 adults (healthy German native speakers, university students, 18–30 years) was recruited. They were either assigned to the Pre-sleep (23.13 ± 0.81 years, *n* = 18, 8 males) or the Sleep condition (23.32 ± 0.53 years, *n* = 19, 10 males), and basically followed the same procedure as the child participants. Participants were part of a larger study and performed another unrelated task, which is reported in more detail elsewhere (Wang et al., 2017). The ethics committee of the local university approved this study.

### Design and Procedures

**FIGURE 1:**
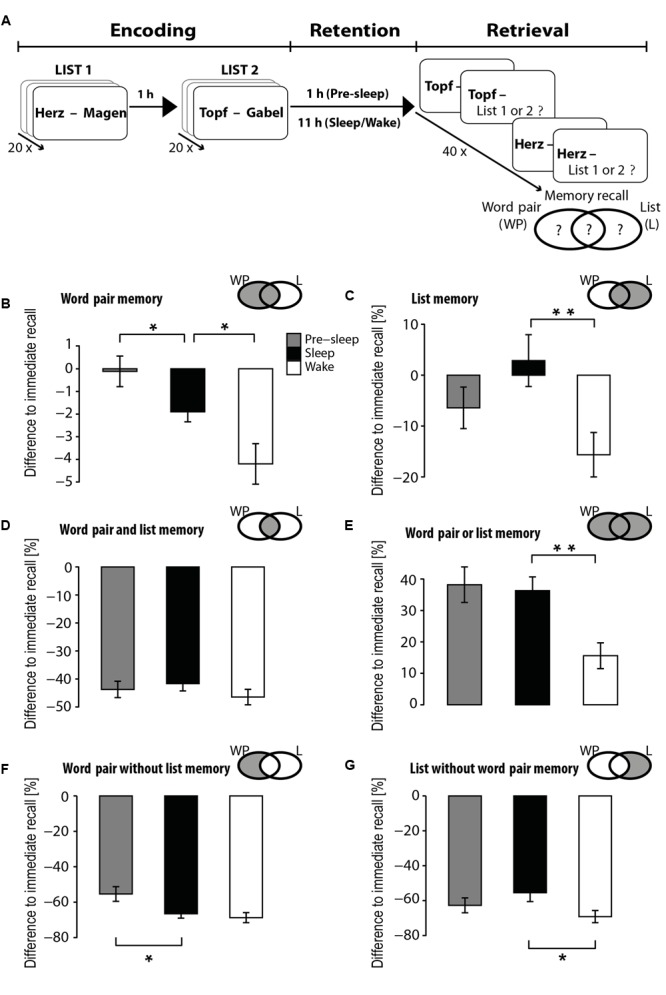
Experimental design and memory dynamics in children. **(A)** The Encoding phase of the experiment consisted of learning two lists of word pairs, each studied once 1 h apart. The duration of the retention interval was either 1 h (Pre-sleep condition) or 11 h with the latter including either a night of sleep (Sleep condition) or daytime wakefulness (Wake condition). In the retrieval phase cued recall was tested for each word pair followed by a recall of the list (forced choice between List 1 and 2) in which the word pair had appeared. Each recall trial was categorized according to the correctness of the associated target word (WP) or the associated list (L) as depicted in the grayed area of Venn diagrams next to the bar graphs. **(B–G)** Children’s mean (± SEM) cued recall performance for the Pre-sleep (gray bars), Sleep (black) and Wake (white) conditions for **(B)** correctly recalled word pairs, **(C)** correctly recalled lists, **(D)** trials with both correctly recalled word pairs and lists, **(E)** trials with correctly recalled word pairs or lists or both, **(F)** trials with correctly recalled word pairs but incorrect list recall, and **(G)** trials with correct list recall but incorrect word pair recall. Recall is expressed as the difference from immediate recall of word pairs during the encoding phase. ^∗^*p* < 0.05, ^∗∗^*p* < 0.01, for pairwise comparisons between retention conditions.

The experimental procedure consisted of an encoding phase, a retention phase, and a retrieval phase. The retention interval was either short (1 h) for children in the Pre-sleep group or long (11 h) in the Sleep and Wake (10 h for the Sleep adults, **Figure 1A**).

Participants of the sleep groups had an adaptation night where they slept in the sleep lab for one night with polysomnographic recordings 1 day before the actual experiments. On the experiment night, children arrived at the lab about 3 h before their usual bedtime. After the preparation for EEG recordings, children encoded two lists of word pairs between 6:00 pm and 8:00 pm with a 1-h break that was filled with standardized lab activities (i.e., playing games with the experimenter). Children went to bed 30 min after completing the encoding phase and slept in the lab for about 9.5 h with polysomnographic recordings. The retrieval phase began 45 min after waking up and consisted of the recall of word pairs and the lists they had appeared in.

The encoding phase of the Wake group children took place between 7:00 am to 9:00 am on the experimental days. After the encoding phase, participants followed their normal daily routine outside the lab. They were asked to avoid stressful mental and physical activities and as well as taking a nap, which was controlled with actigraphy (Actiwatch 2, Philips, Netherlands). After a retention interval of about 11 h, participants came back to the lab to complete the retrieval phase at around 7:00 pm.

Children in the Pre-sleep group came to the lab for the experimental evening about 3.5 h before their usual bedtime, and the encoding phase took place between 4:00 pm and 6:00 pm. The retrieval phase took place 1 h after the encoding phase was completed. Control analyses performed to exclude possible confounding influences of vigilance are described in detail in the Supplementary Material.

### Word Pair Learning

The paired-associate learning task comprised 40 (80 for adults) semantically related word pairs that had been used in a previous study showing a clear sleep benefit for word pair learning in children and adults (Wilhelm et al., 2008). In the present study, these word pairs were split randomly into two equally sized lists with the word pairs in a random order to introduce a clear spatiotemporal context difference during encoding (lists studied 1 h apart). Instead of being read aloud by an experimenter like in the study by Wilhelm et al. (2008), word pairs were presented on a PC screen for 6 s with 1 s pause for children and 4 s with 1 s pause for adults, respectively. Participants were instructed to remember the word pairs for later recall (item memory). No instructions were given to remember the temporal context (list order), however, word pair lists were introduced as “List 1” or “List 2” on the PC screen. Children had one opportunity to encode the list to stress the episodic nature of the task (one-time learning) instead of repeated learning until a certain criterion was reached (as in previous studies). Right after encoding of each list, children were shown one cue word of each word pair on the PC screen and were asked to orally recall the corresponding target word without any feedback (immediate recall). After the retention phase, delayed recall of word pairs from both lists was tested in a random order. Additionally, participants had to indicate in which of the two lists a specific word pair (item) had been presented originally (source memory).

The memory task was designed for one-time encoding, thus precluding the exclusion of poor performers right away. Therefore, poor performers with an average immediate word pair recall below 40% were excluded from the analyses (Children: *n* = 5, adults: *n* = 4).

### Sleep Recordings and EEG Analysis

Sleep was recorded using standard polysomnography including EEG recordings from Fz, F3, F4, Cz, C3, C4, Pz, P3, and P4 electrode sites (reference: linked electrodes at the mastoids, ground: Fpz), electromyography on the chin (musculus mentalis), and electrooculography (around the eyes). Signals were amplified (BrainAmp, Brain Products, Gilching, Germany), digitized (sampling rate > 250 Hz) and filtered (EEG and EOG 0.3–35 Hz, EMG 10–100 Hz). Sleep stages were scored offline by two experienced raters according to standard criteria (Rechtschaffen and Kales, 1968). General spindles (9–15 Hz) as well as fast and slow spindles were detected using standard settings of the SpiSOP tool (Weber, 2016) which was based on previously published algorithms (Mölle et al., 2002). Spindle parameters (e.g., density) were averaged across the anterior-posterior axis, i.e., Fz, Cz, and Pz. Fast and slow spindles were detected in a 2-Hz frequency band centered to the visually determined corresponding power peak in the Non-REM power spectrum of each participant (Wang et al., 2017). Supplementary Table S1 contains a summary of the sleep scoring and spindle parameters as well as correlations with overnight changes in each memory measure (uncorrected for multiple comparisons).

### Statistical Analysis

Statistical analysis was done using [*R*] (Mac OS X version 1.7.1, R Core Team, 2012). Mean ± SEM are reported. Kruskal–Wallis one-way ANOVA was used as non-parametric test in case normality and homogeneity assumptions of ANOVA were not met. *Post hoc* tests followed significant ANOVAs effects, including Student’s *t*-test for equal variances and Welch’s *t*-test with approximation to the degrees of freedom for unequal variances; otherwise we used non-parametric Mann–Whitney *U* test. Cohen’s *d* indicated central effect sizes. Moreover, associations were tested using linear regression analysis with Pearson product-moment and Spearman’s rank correlation for the parametric and non-parametric tests, respectively. For simplicity, *p*-values are reported uncorrected for multiple comparisons. Significance level was set to 0.05.

## Results

Immediate recall of word pairs in the children neither differed between the three retention groups [Pre-sleep, Sleep, Wake, *F*_(2,50)_ = 0.89, *p* > 0.4] nor between List 1 and 2 [*F*_(1,50)_ = 0.85, *p* > 0.4]. Forgetting dynamics over the retention interval (measured as the difference in the number of correct word pairs at delayed recall from the number of correct word pairs at immediate recall) were, however, markedly different between retention groups [*F*_(2,50)_ = 8.96, *p* < 0.001, one-way ANOVA]. Across the short 1-h retention interval (Pre-sleep), forgetting of word pairs were virtually absent. Forgetting across the longer 11-h interval was smaller in the Sleep group compared to the Wake group [*t*_(29.72)_ = 2.22, *d* = 0.73, *p* = 0.03 for Pre-sleep vs. Sleep; *t*_(31)_ = 3.71 *d* = 1.28, *p* < 0.001 for Pre-sleep vs. Wake; *d* = 0.82, *t*_(20.71)_ = 2.30, *p* = 0.03 for Sleep vs. Wake, two-tailed *t*-tests, **Figure 1B**].

The absolute number of word pairs with correct list recall (irrespective of whether the word pair was recalled correctly or not) was comparable across conditions [Pre-sleep: 22.72 ± 0.97, Sleep: 24.00 ± 0.78, Wake: 22.07 ± 0.78, *F*_(2,50)_ = 1.33, *p* = 0.27], and there was no significant difference between list recall for the Sleep and Wake groups [*t*_(33)_ = -1.72, *p* = 0.095]. Because encoding performance as a measure for general memory performance could already explain some of the observed inter-individual differences in list recall (see below) we also expressed list recall as the difference from the individual’s word pair recall at the immediate recall test (which was set to 100%) serving as an (approximate) baseline. Indeed, this measure revealed a pronounced enhancing effect of sleep vs. wakefulness on list recall [*F*_(2,50)_ = 3.89, *p* = 0.03; *t*_(33)_ = 2.64, *p* = 0.01, for Sleep vs. Wake group, **Figure 1C**].

To disentangle the effects of sleep on word pair recall and list recall, we separately analyzed the effects for recall trials (i) on which word pair recall *or* list recall was correct (**Figure 1E**), further subdivided into the three categories: (ii) trials on which both word pair recall *and* list recall was correct (memory for the item bound into its source, note that we cannot distinguish if the source memory was bound to the cue or target or both words in this case, **Figure 1D**), (iii) trials on which only word pair recall was correct but not list recall (**Figure 1F**), and (iv) trials on which only list recall but not word pair recall was correct (**Figure 1G**), with all of the measures adjusted to the individual’s immediate word pair recall (see Supplementary Figure S1 for the absolute number of recall trials by subcategory for each experimental group). Note that such baseline adjusted values do not imply improvements or decreases in memory over the retention interval for the list recall measures (i.e., for categories i, ii, and iv). Unexpectedly, sleep did not significantly enhance combined recall of the word pair together with the list the word pair had appeared in [i.e., the item bound into its source, *F*_(2,50)_ = 0.73, *p* = 0.49, for main effect Condition; *t*_(33)_ = -1.25, *p* = 0.22, for Sleep vs. Wake group, **Figure 1D**]. By contrast, a large beneficial sleep effect was revealed for general recall of either the word pair or the list or both [i.e., any of item or source, bound and unbound, *F*_(2,50)_ = 6.13, *p* = 0.004; *t*_(33)_ = -3.35, *p* = 0.002, for Sleep vs. Wake group, see **Figure 1E**]. This sleep effect did not appear to be driven by correct word pair recall for which list recall was incorrect (i.e., separate word pair recall, *p* = 0.56, for Sleep vs. Wake group, **Figure 1F**), but rather by trials with correct list recall but incorrect recall of the target word [i.e., separate list recall, *t*_(31.71)_ = -2.24, *p* = 0.032, for Sleep vs. Wake, **Figure 1G**].

Forgetting from the short 1-h retention interval (Pre-sleep) to the long 11-h retention intervals (Sleep, Wake) occurred at a significant level only for the trials with correct word pair recall but incorrect list recall (*p* = 0.023 and *p* = 0.016 for comparison with Sleep and Wake, respectively, **Figure 1F**). Forgetting was not significant for trials with only correct list recall (both *p*s > 0.26, **Figure 1G**).

Correlational analyses did not reveal any strong and significant association between delayed recall of word pairs and list recall in any of the groups (all *r*s < 0.36, all *p*s > 0.14), indicating that – in all experimental groups – both types of recall were largely independent at delayed recall. Furthermore, correlational analyses revealed that word pair recall at the immediate recall test was associated with later list recall across the three retention conditions (*r* = 0.27, *p* = 0.048) suggesting these measures share a component of “general memory capabilities.” Such shared components can be taken to justify our use of immediate word pair recall values (as an estimate of memory encoding) for adjusting the individual’s list recall in the homogeneous group of children (see above).

### Correlations between Memory Performance and Sleep Parameters

Sleep in children showed the expected pattern with longer overall duration and remarkably greater amounts of slow-wave sleep than adults (Supplementary Table S1). Of the correlations calculated between sleep parameters and memory performance, only a few remained significant after correcting for multiple testing (false discovery rate). Combined recall of word pairs and their respective list (adjusted to the individual’s encoding performance) correlated positively with the percentage of SWS (*r* = 0.61, *p* = 0.009) and negatively with the percentage of Stage 2 sleep (*r* = -0.61, *p* = 0.01). Spindle density during Non-REM sleep (detected in the 9–15 Hz range) correlated negatively with general word pair memory (*r* = -0.59, *p* = 0.012) and recall of word pairs without correct list recall (*r* = -0.58, *p* = 0.014, see Supplementary Material for details on the full exploratory correlation analysis in Supplementary Table S1, including additional separate analyses for fast and slow spindle parameters associated with each memory type, uncorrected for multiple comparisons).

### Comparison of the Temporal Dynamics of Memory between Children and Adults

To explore if the forgetting dynamics across the short 1-h and long 11-h retention intervals in children differed from those in adults, we tested two groups of adults on the respective Pre-Sleep and Sleep conditions. To account for differences in general learning capabilities between children and adults, we used longer lists in the adults and age groups were compared based on the percentages of recalled word pairs with reference to the total number of word pairs per list (see Materials and Methods). Also, to ease interpretation of the list recall measures and to account for the observed differences in general learning capabilities in children and adults, we refrained from adjusting list recall measures after 1 and 11 h to immediate word pair recall performance in respective sub-analyses.

**FIGURE 2:**
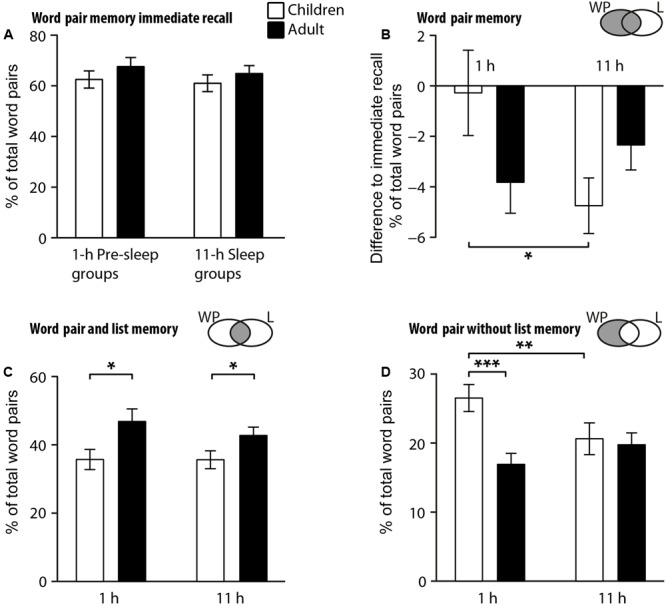
Comparison of recall performance between children (white bars) and adults (black) for the 1-h Pre-sleep condition (left bars) and 11-h Sleep conditions (right bars) for **(A)** immediate recall of word pairs during the encoding phase **(B)** delayed recall of word pairs (expressed as the difference from immediate recall as in **A**), and for subgroups of trials **(C)** with combined correct word pair and list recall and **(D)** with correct word pair but incorrect list recall. Recall is expressed as the percentage of the total number of word pairs presented at the encoding phase (40 in children, 80 in adults). Note, recall in **(C,D)** is not adjusted to the individual’s immediate recall during the encoding phase. Gray area in the Venn diagrams next to the bar graphs depicts the trial category as in **Figure 1** with respect to correctness of the delayed word pair (WP) or associated list (L) recall. ^∗^*p* < 0.05, ^∗∗^*p* < 0.01, ^∗∗∗^*p* < 0.001 for pairwise comparisons.

Indeed, the percentage of recalled word pairs at immediate recall did not differ between age and retention groups (*p* = 0.19 for the main effect of Age, and *p* = 0.54 for the main effect of Pre-Sleep vs. Sleep group, **Figure 2A**). Children showed no forgetting of word pairs at the 1-h recall and increased forgetting after 11 h (*p* = 0.034), whereas adults showed substantial forgetting already at the 1-h recall with no further increase at the 11-h recall [*F*_(1,67)_ = 5.29, *p* = 0.025, for Age × Pre-sleep/Sleep interaction, **Figure 2B**]. Notably, in the sub-analyses this differential forgetting dynamics in children was only present for the trials with separate word pair recall without correct list recall [*F*_(1,67)_ = 4.94, *p* = 0.03, for Age × Pre-sleep/Sleep, **Figure 2D**] but not in any other subgroup of trials, including the trials with combined correct word pair and list recall (*p* > 0.49, **Figure 2C**). In fact, the percentage of trials with combined correct word pair and list recall was generally smaller in children than adults [*F*_(1,67)_ = 9.45, *p* = 0.003, **Figure 2C**], and the number of trials with separate correct word pair recall without correct list recall was generally higher in children than adults [*F*_(1,67)_ = 6.97, *p* = 0.01, Age main effects].

## Discussion

We used a modified version of the word paired-associate learning task to determine the extent to which sleep’s enhancing effect on word pair memories in children might originate from sleep specifically strengthening episodic features of the memory representations. Compared to wakefulness, post-learning sleep enhanced word pair recall, in general, which replicates several previous studies in children (Backhaus et al., 2008; Wilhelm et al., 2008; Potkin and Bunney, 2012) and underlines the robustness of the effect that emerged despite the necessary task changes introduced compared to other studies (one-time encoding of word pairs, encoding of two different lists 1 h apart). Sleep also benefited general list memory as well as isolated list memory (i.e., trials with correct list recall but incorrect word pair recall). Surprisingly, however, no sleep benefit was revealed for the combined word pair and list memory.

Assuming that the combined word pair and list memory is a measure closely reflecting the item-source binding characteristic of episodic memory, the absence of any enhancing effect of sleep on this measure argues against the view that sleep effects on episodic features of the memory essentially contribute to the general enhancement in word pair memory, all the more so since both measures of memory performance were uncorrelated. The absence of a sleep-induced enhancement in combined word pair and list recall also diverges from previous findings indicating a sleep-induced enhancement of episodic “what-where-when” memory in children of the same age group, although in that study the gain in episodic memory after sleep was not superior to that seen in adults (Wang et al., 2017). A tentative explanation for this discrepancy is that the previous study manipulated spatial as well as temporal context aspects of the episode, whereas source memory was mainly defined by the temporal context aspects here, i.e., by the second list learned 1 h after the first list. Temporal features of episodic memory formation show a protracted development well into adolescence (e.g., Picard et al., 2012). Another explanation for sleep having no impact on the combined word pair and list memory is that these may have been the strongly encoded memories known to benefit less from sleep than weakly encoded memories (Diekelmann et al., 2009; Wilhelm et al., 2012).

Indeed, the findings of absent or only moderate sleep-induced enhancements in measures of episodic memory in this and previous studies might simply reflect the immaturity of the episodic memory system and the fact that at this age children’s encoding and forming memories for episodes is less well structured in time and space (Ghetti and Bunge, 2012; Riggins, 2014). Supporting this view, the comparison of memory dynamics with an adult control group revealed generally reduced memory for word pairs in combination with the correct list, but enhanced memory for word pairs in the absence of correct list recall, i.e., children appear to preferentially store word pair memories unbound to their source. On the other hand, correlational analyses confirmed that like in adults (Inostroza and Born, 2013), SWS in children preferentially supports episodic-like memory, here of combined memory for the word pair and the list it appeared in. In this context, the strong negative correlation of EEG spindle density with separate word pair memory (in the absence of correct list recall) and with general word pair memory, was unexpected and also diverges from findings in adults of a link between spindles and non-episodic semantic types of memory (e.g., Schabus et al., 2004). However, in children, higher spindle density has been associated with poorer reading ability (Bruni et al., 2009) suggesting that more spindles might not necessarily indicate better learning or consolidation in all cognitive domains in children. This might point to differential functions of sleep spindles for memory processing in children. It remains unclear to what degree these associations relate to children’s unique sleep architecture. Indeed the fine-tuned synchronization between sleep spindles and slow oscillations seem instrumental to consolidating hippocampal dependent memories (Staresina et al., 2015; Greenberg et al., 2016; Maingret et al., 2016) including word pair memories (Niknazar et al., 2015). It may be that spindles and slow-oscillations in children are not yet fine-tuned to reflect the associations between spindles and consolidation of the mature brain.

Apart from enhancing general word pair memory, sleep in the children also generally enhanced list memory, as well as isolated list memory (in the absence of correct word pair memory). The result of particularly strong effects of sleep on isolated list memory is a further hint that sleep in children does not act toward enhancing episodic memory features (binding source to item characteristics). In fact, sleep-induced enhancements of word pair and list memories that are entirely independent of whether or not respective source or item information is also correctly recalled, could be taken to speculate that sleep in children fosters the “unbinding” of item and source information in episodic representations. Note, however, that our data does not show that sleep reduces item-source bound memory (i.e., “unbinding”) but rather suggests that sleep leaves it unaffected. Sleep “unbinding” episodic representations has been observed in adults although not consistently and often developing more slowly over several nights (Cairney et al., 2011; Deliens and Peigneux, 2014; but see Sweegers and Talamini, 2014; Jurewicz et al., 2016). That children form less distinct episodic memory overall might lead to faster unbinding effects in children. This might also explain the strong enhancement of sleep on list memory considered incidentally encoded source information, as encoding in children would be expected to be less distinct between source and item information. However, the hypothesis of a fast unbinding effect of sleep on episodic memory in children, although attractive, needs to be scrutinized using task designs directly testing the context-dependency of item recall after sleep.

## Ethics Statement

This study and its protocol was approved and carried out in accordance with the recommendations of the ethics committee of the Medical Faculty of the University and University Clinics Tübingen. All subjects gave written informed consent in accordance with the Declaration of Helsinki.

## Author Contributions

J-YW and FW designed the study. J-YW, FW, KZ, and JB wrote the manuscript. HN provided critical revisions on an earlier version of the manuscript. J-YW and FW conducted the study and analyzed the data.

## Conflict of Interest Statement

The authors declare that the research was conducted in the absence of any commercial or financial relationships that could be construed as a potential conflict of interest.
