# Supplementary material for: Effects of Sleep on Word Pair Memory in Children – Separating Item and Source Memory Aspects
Source: Front Psychol. 2017 Sep 7;8:1533. doi: 10.3389/fpsyg.2017.01533 (PMC5594220; doi:10.3389/fpsyg.2017.01533)
Supplement: Supplementary file 1 [file Data_Sheet_1.DOCX]

Supplementary Material

Effects of Sleep on Word-Pair Memory in Children – Separating Item and Source Memory Aspects

Jing-Yi Wang, Frederik D. Weber, Katharina Zinke, Hannes Noack, Jan Born^*^

*** Correspondence:**Jan Born

Institute of Medical Psychology and Behavioral Neurobiology

University of Tübingen

Otfried-Müller-Str. 25

72076 Tübingen

Germany

jan.born@uni-tuebingen.de

Tel: +49(0)7071-29-88924

Fax: +49(0)7071-29-25016

# Sleep parameters and correlation with memory measures

Supplementary Table 1 contains a summary of the sleep scoring and spindle parameters as well as correlations with overnight changes in each memory measure (uncorrected for multiple comparisons). Due to exclusion of poor performers (see Methods) correlation analysis of sleep scoring, spindle and slow oscillation parameters with overnight changes in memory (as presented in Figure 1) included one Sleep group child less than was usable from the EEG sleep recordings (n = 17). Recall of word pairs with simultaneously correct list recall (adjusted to the individual’s encoding performance) correlated positively with the percentage of SWS and negatively with the percentage of Stage 2 sleep. Spindle density during Non-REM sleep correlated negatively with general word-pair memory and recall of word pairs without correct list recall. Of note, spindle count displayed the reverse association with correct list recall and correct list without word-pair recall maybe suggesting differential roles of spindles for word-pair (item) and list (source) memory consolidation.

**Supplementary Table 1**

*Sleep parameters and correlations with word-pair and list memory changes in children*

| **Sleep parameter (*n* = 18)** | | | | **Correlations with memory recall [*r*] (n = 17)** | | | | | | | | |
| --- | --- | --- | --- | --- | --- | --- | --- | --- | --- | --- | --- | --- |
| **Stages** | | | | **WP** | | **L** | **WP & L** | | **WP or L** | **WP w/o L** | | **L w/o WP** |
| TST [min] | 553.19 | ± | 3.45 | -.25 | | .20 | -.32 | | .30 | .12 | | .37 |
| Sleep onset [min] | 15.80 | ± | 3.97 | .36 | | -.13 | .46 | | -.26 | -.21 | | -.36 |
| WASO [%] | 0.25 | ± | 0.16 | **-.56*** | | -.17 | .09 | | -.06 | -.46 | | .13 |
| Stage 1 [%] | 2.07 | ± | 0.18 | -.01 | | -.04 | -.37 | | .16 | .36 | | .15 |
| Stage 2 [%] | 21.66 | ± | 1.61 | -.26 | | -.26 | **-.61**** | | -.07 | .43 | | .04 |
| SWS [%] | 49.89 | ± | 1.97 | .26 | | .29 | **.61**** | | .09 | -.45 | | -.01 |
| Non-REM [%] | 71.56 | ± | 1.04 | .05 | | .08 | .08 | | .05 | -.09 | | .04 |
| REM [%] | 25.07 | ± | 0.86 | .07 | | -.08 | -.05 | | -.02 | .14 | | -.06 |
| **Spindles** in Non-REM | | | | | | | | | | | | |
| Count | 1303 | ± | 45 | **-.55*** | **.50*** | | | .05 | .30 | | **-.50*** | **.49*** |
| Density [1/min] | 3.80 | ± | 0.10 | **-.59*** | .45 | | | .12 | .20 | | **-.58*** | .40 |
| **Slow spindles** in Non-REM | | | | | | | | | | | | |
| Count | 1380 | ± | 54 | -.41 | .45 | | | .37 | .13 | | **-.71**** | .28 |
| Density [1/min] | 4.02 | ± | 0.14 | -.33 | .37 | | | **.52*** | -.02 | | **-.79***** | .11 |
| **Fast spindles** in Non-REM | | | | | | | | | | | | |
| Count | 1623 | ± | 46 | **-.59*** | .41 | | | .13 | .14 | | **-.58*** | .36 |
| Density [1/min] | 4.74 | ± | 0.11 | **-.61**** | .26 | | | .26 | .09 | | **-.70**** | .14 |

Means ± SEM for the sleep parameters in the children are shown in the left columns. The right columns show Pearson’s correlation of sleep parameters with overnight changes in memory as presented in Figure 1, i.e. word-pair memory (WP), list memory (L), word-pair and list memory (WP & L), word-pair or list memory (WP or L), word-pair without list memory (WP w/o L) and list without word-pair memory (L w/o WP). Given are the total sleep time (TST), sleep onset (with reference to the time of lights off and beginning of first occurrence of stage 1-sleep epoch followed by stage 2-sleep), and time spent awake after sleep onset (WASO), sleep stage 1, sleep stage 2, SWS, Non-REM (S2 + SWS) and REM in percentage of total sleep time. In addition, general spindle (9–15 Hz), fast and slow spindle parameters (absolute count and density per minute) averaged across the anterior-posterior axis, i.e., Fz, Cz and Pz. Significant correlations are in bold, *** *p < .*001, ** *p < .*01, * *p < .*05, uncorrected for multiple comparisons.

# Absolute number of trials in each subcategory of the memory measure


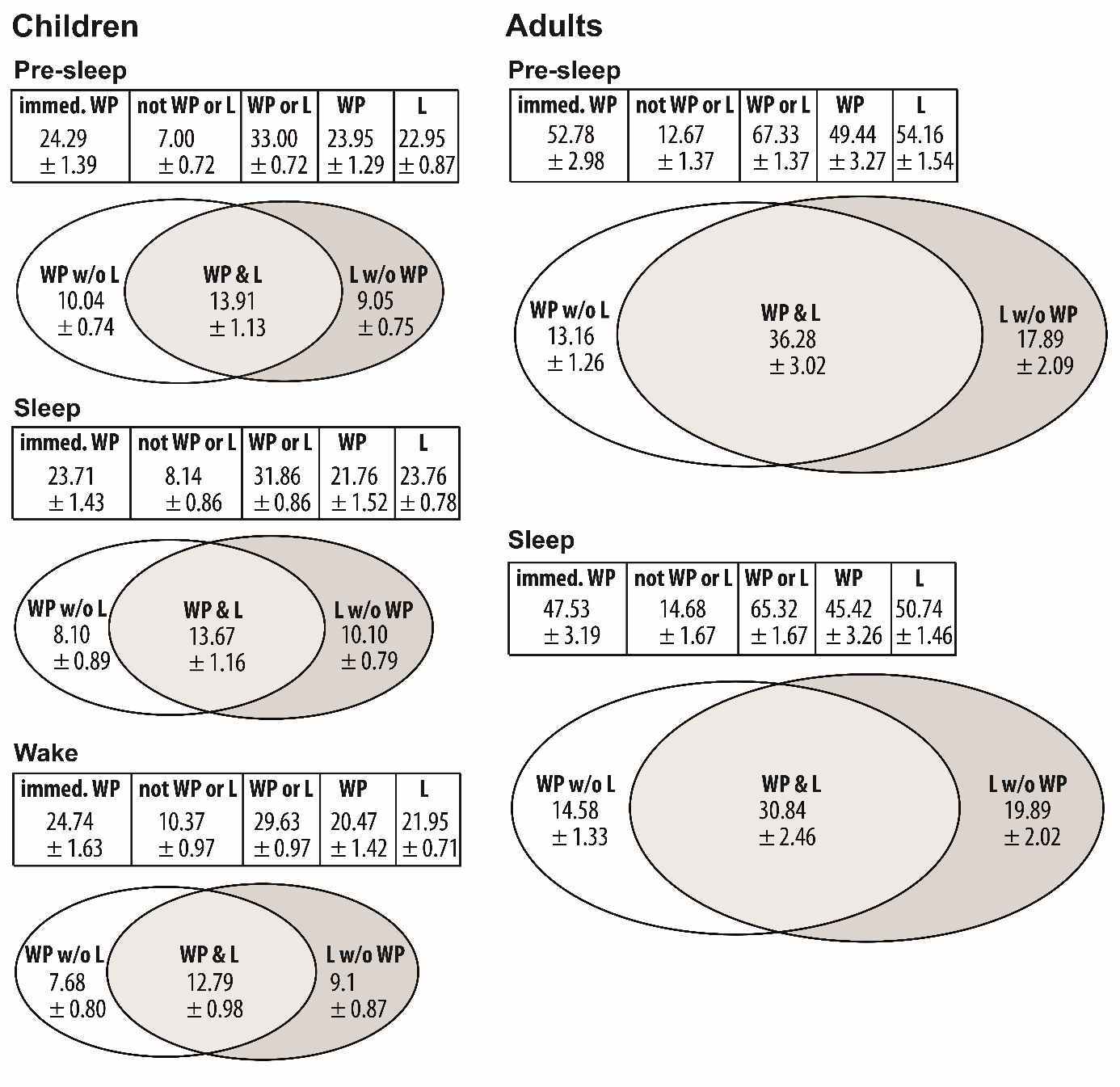


**Figure S1**: Means ± SEM of trials in each of the recall categories for children and adults and each condition, separately. Note that adults initially learned twice as many word pairs as children. Values in the small tables above the Venn diagram (overlapping circles) give the number of trials in the immediate recall on only the word-pair memory (immed. WP) and the delayed recall with the trials that were correct for word-pair or list memory (WP or L) or not (not WP or L), as well as the word-pair memory (WP) and the list memory (L). The areas of the left and right circles in the Venn diagram are sized according to the absolute number of trials for delayed recall of word-pair memory (WP) and list memory (L), respectively. The numbers inside the Venn diagrams subdivide those trials into the trial categories of word-pair without list memory (WP w/o L), word-pair and list memory (WP & L), and list without word-pair memory (L w/o WP) with the areas adjusted to the proportion of trials in each of those categories.

# Children’s vigilance and subjective tiredness

Before the encoding of each list and before retrieval vigilance was assessed using reaction time performance during a 5-min version of the Psychomotor Vigilance Task (PVT). Furthermore, subjective tiredness was assessed using the Stanford Sleepiness Scale (SSS, Hoddes, Dement, & Zarcone, 1972).

Reaction time on the PVT did not differ between Pre-sleep, Sleep, and Wake conditions, neither at encoding (Pre-sleep: 403.56 ± 13.84 ms, Sleep: 420.64 ± 33.44 ms, Wake: 390.21 ± 15.27 ms) nor at retrieval (Pre-sleep: 449.04 ± 32.48 ms, Sleep: 391.35 ± 16.03 ms, Wake: 408.69 ± 19.44 ms, all *p* > .22). Subjective tiredness (as assessed by the SSS) of the Pre-sleep group was lower than the other two groups at encoding (Pre-sleep: 1.58 ± 0.14, Sleep: 2.63 ± 0.30, Wake: 2.61 ± 0.30, *p* < .007 for both comparisons) but did not differ between the groups at retrieval testing (Pre-sleep: 1.78 ± 0.17, Sleep: 2.41 ± 0.26, Wake: 2.36 ± 0.34, *p* > .19). Considering SSS at retrieval or encoding as covariate did not essentially change any of the reported ANOVA effects for memory recall except that the decrease in general word-pair memory from the Pre-sleep to the Sleep condition in children failed to reach significance (Figure 2B). This suggests the reduced forgetting in the Pre-sleep children might be partially driven by lower tiredness at encoding in this condition. Otherwise, these results exclude that sleep-wake related differences in memory recall were confounded by non-specific alterations in vigilance.

**References**

Hoddes, E., Dement, W.C., & Zarcone, V. (1972). The development and use of the Stanford Sleepiness Scale. *Psychophysiology*.
